# Multi-Stream Convolution-Recurrent Neural Networks Based on Attention Mechanism Fusion for Speech Emotion Recognition

**DOI:** 10.3390/e24081025

**Published:** 2022-07-26

**Authors:** Huawei Tao, Lei Geng, Shuai Shan, Jingchao Mai, Hongliang Fu

**Affiliations:** College of Information Science and Engineering, Henan University of Technology, Zhengzhou 450001, China; genglei202207@163.com (L.G.); shanshuai1313@163.com (S.S.); mjc_haut@163.com (J.M.); jackfu_zz@163.com (H.F.)

**Keywords:** speech emotion recognition, feature extraction, hybrid neural network, multi-head attention mechanism, feature fusion

## Abstract

The quality of feature extraction plays a significant role in the performance of speech emotion recognition. In order to extract discriminative, affect-salient features from speech signals and then improve the performance of speech emotion recognition, in this paper, a multi-stream convolution-recurrent neural network based on attention mechanism (MSCRNN-A) is proposed. Firstly, a multi-stream sub-branches full convolution network (MSFCN) based on AlexNet is presented to limit the loss of emotional information. In MSFCN, sub-branches are added behind each pooling layer to retain the features of different resolutions, different features from which are fused by adding. Secondly, the MSFCN and Bi-LSTM network are combined to form a hybrid network to extract speech emotion features for the purpose of supplying the temporal structure information of emotional features. Finally, a feature fusion model based on a multi-head attention mechanism is developed to achieve the best fusion features. The proposed method uses an attention mechanism to calculate the contribution degree of different network features, and thereafter realizes the adaptive fusion of different network features by weighting different network features. Aiming to restrain the gradient divergence of the network, different network features and fusion features are connected through shortcut connection to obtain fusion features for recognition. The experimental results on three conventional SER corpora, CASIA, EMODB, and SAVEE, show that our proposed method significantly improves the network recognition performance, with a recognition rate superior to most of the existing state-of-the-art methods.

## 1. Introduction

Speech is the most basic and effective way of communication. It contains not only content information, but also emotional information. People can perceive the emotional changes of other individuals from speech. Meanwhile, speech is also the most important communication mode of human–computer interaction. So, speech emotion recognition is the most important research direction in the future generation of human–computer interactive speech systems [1,2,3,4], and conducting research on this topic is of both great theoretical and practical value.

In order to build a speech emotion recognition system with excellent performance, scholars have undertaken an in-depth exploration from the aspects of feature extraction, feature fusion, and so on. Traditional speech acoustic features mainly include prosodic features, voice quality features, and spectrum-based features. D.H. Milone et al. [5] presented an analysis of prosody’s three most important parameters, namely energy, fundamental frequency, and duration, together with a method for incorporating this information into automatic speech recognition. Using this approach obtained a word recognition error reduction rate of 28.91% with a Spanish continuous-speech database. S Zhang et al. [6], utilizing the support vector machine classifier, discriminated four emotions from a Chinese natural emotional speech corpus, including anger, joy, sadness, and neutral, by combining prosody and voice quality features. The experiment results show an approximately 10% improvement in accuracy rate compared with using the single prosody features. Y Sun et al. [7] present novel weighted spectral features based on local Hu moments. To describe the local energy distribution of a spectrogram, Hu moments computed from local regions of spectrogram are used. The conducted experiments validate the proposed features in terms of the effectiveness of the speech emotion recognition. The above research results have been widely used in the classical speech emotion recognition system.

In recent years, deep learning technology has shown extremely excellent performance in the field of speech emotion recognition. Many neural network models are widely used in emotion recognition, including the convolutional neural network (CNN) [8,9], deep neural network (DNN) [10], auto-encoder network (AE) [11,12], long and short-term memory network (LSTM) [13,14], ladder network [15], etc.

In order to explore the potential performance advantages of CNN in a speech emotion recognition system, Q Mao et al. [8] used a CNN and AE network to extract speech emotion features and demonstrated the feasibility of CNN in speech emotion recognition. In view of the problem of insufficient labeled speech emotion data, S Zhang et al. [9] used the AlexNet framework trained by the Imagenet data set to build a deep learning network, fine tuning the network with a speech spectrum, and completing the construction of a deep convolutional neural network (DCNN). This method solves the problem of insufficient label data to some extent. S Kwon et al. [16] proposed a two-stream deep convolutional neural network with an iterative neighborhood component analysis to learn mutually spatial-spectral features and select the most discriminative optimal features for the final prediction. The research shows that constrained by the few number of samples, when the deepening of network layers, CNN’s representation of global emotional features is gradually disordered [17], and the information entropy of emotional features increases. In order to solve this problem, researchers used LSTM network for speech emotion recognition. Using the powerful temporal emotion information modeling ability of LSTM for reference, the temporal emotion features in speech are extracted. Yue X et al. [13] used LSTM in speech emotion recognition and extracted frame level features from speech as a feature input of the LSTM network, applying the attention mechanism in time and feature dimensions and improving the emotional representation ability of the LSTM network. At the same time, scholars composed CNN and LSTM into a complex and effective hybrid neural network, taking the temporal emotional features learned by LSTM as a supplement to the global emotional features lost by CNN, e.g., as M Chen et al. [18] designed the convolutional-recurrent neural networks integrating DCNN and LSTM networks. Fang et al. [19] designed a hybrid network integrating DCNN and AE network Compared with a single network, hybrid networks can obtain more emotional information and achieve better recognition ability.

Although the hybrid neural network has enabled many achievements, the above research does not fundamentally solve the problem that the global emotional features extracted by a CNN are gradually disordered due to the increase of information entropy, and there is a lack of in-depth research on feature fusion. In ref. [20], different network features are directly spliced to achieve feature fusion, which cannot obtain the optimal feature set. In refs. [21,22], the author realizes classification fusion from decision-level fusion. In ref. [19], Fang et al. used the attention mechanism to realize feature fusion, but it is only limited to two categories and has not been involved in multi category classification.

**Figure 1:**
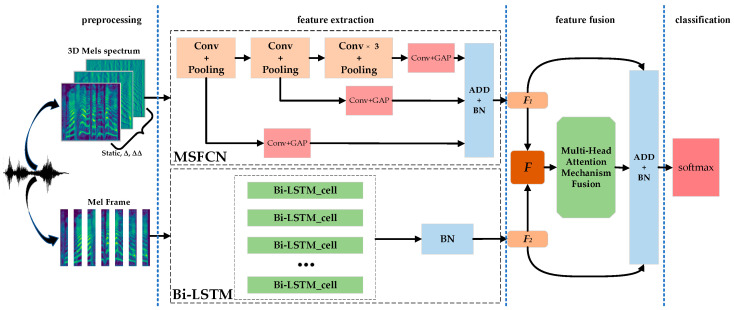
MSCRNN-A model structure.

Based on the above analysis, we propose a new SER method, which aims to use the multi-stream sub-branches convolution neural network structure to solve the problem of global emotional feature loss from the perspective of CNN itself, and to realize the adaptive fusion of multi network features by using the multi head attention mechanism to improve the performance of a set task. Figure 1 shows the overview architecture of our proposed method. In this architecture, we design a parallel network model to extract different emotional features. In order to reduce the information loss in convolutional networks, we add subbranches after all pool layers. Then, a multi-attention mechanism is used to calculate the weight of different features, and different features are weighted adaptively. The output of a different network is connected with the output of the multi-head attention mechanism feature weighted layer through shortcut connection to realize feature fusion. Finally, emotion recognition is realized by Softmax.

The contribution of this paper is as follows.

(1) A new multi-stream sub-branch full convolution network (MSFCN) for emotion recognition is proposed, which is based on AlexNet. The loss of global emotion information can be effectively reduced by adding branches.
(2) A feature fusion method based on the multi-head attention mechanism is proposed, which realizes feature adaptive weighting. In particular, the feature fusion method proposed by this paper can be carried out at the same time with network parameter adjustment, so as to obtain the optimal feature set.
(3) A new speech emotion recognition method is constructed, which can achieve excellent recognition results on different emotion recognition databases.

## 2. Methods

The proposed method is shown in Figure 1, including: preprocessing, feature extraction, feature fusion, classification. In the preprocessing, the speech data are processed to produce *Mel* spectrum and *Mel* frames, which are used as the input of MSFCN and Bi-LSTM respectively. In order to make the model learn the spatial structure and temporal dependence of emotional information in the speech *Mel* spectrum at the same time, MSFCN and Bi-LSTM modules are set in parallel in the feature extraction stage, in which the MSFCN module focuses on the capture of time-frequency information in the spectrum, and Bi-LSTM module is responsible for extracting the temporal information of the spectrum. In MSFCN, Conv + Pooling, 1 × 1 Conv, GAP, and ADD + BN represent the convolution and pooling layer, 1 × 1 convolution layer, global average pooling, additive summation, and batch normalization. The feature fusion stage is supposed to obtain the best fusion feature of the two features of the parallel network output, which mainly consists of the multi-head attention mechanism adaptive weighting layer, shortcut connection, dropout, and + BN layer.

### 2.1. Data Input

Feature extraction is the most important part of speech emotion recognition, determining the performance of an emotion recognition system. In recent years, *Mel* spectrum has been widely used in emotion recognition because it contains a large amount of emotional information [8,9,17,18]. In view of this, this paper uses *Mel* spectrum data as the input of the network. As shown in Figure 1, the MSFCN network is used to extract the global emotional information of *Mel* spectrum, and the Bi-LSTM network is used to extract the continuous frame features of *Mel* spectrum, so as to realize the complementary of different network features.

For each speech, a 64 channel *Mel* spectral filter with 25 ms Hamming window and 10 ms frame shift is used to process the speech. Then, the frame data $Frames\in R^{T\times 64}$ are obtained, where T represents the number of frames for speech and 64 for the number of *Mel* filters. $Frames\in R^{T\times 64}$ becomes the input of Bi-LSTM.

As MSFCN needs image as input, all the frame data of each speech are spliced together to form the *Mel* spectrum Mel. For dynamic emotional information, the first and second derivative of Mel is calculated to obtain ΔMel and ΔΔMel. With Mel, ΔMel, and ΔΔMel, as three color channels of an image, a color picture, $Mels\in R^{T\times F\times 64}$, is formed. Considering that the emotional speech is not of equal length, it is impossible to input Mels directly into the MSFCN network. Therefore, this paper uses a bilinear difference algorithm to change the size of Mels to adapt to the network input.

### 2.2. Multi-Stream Sub-Branches Full Convolution Network

Refs. [5,9,11] have proven that the AlexNet network has good speech emotion recognition performance. At the same time, AlexNet’s smaller parameter scale is conducive to balance the computation between the convolutional network and Bi-LSTM. However, the emotional corpus contains a relatively few samples, with the deepening of network layers, the problem of feature loss will appear. Therefore, a multi-stream sub-branch full convolution network based on AlexNet is proposed to reduce the loss of emotional information.

**Figure 2:**
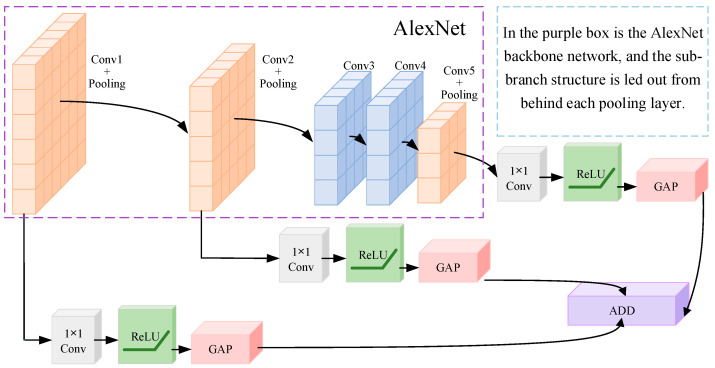
MSFCN model structure.

First, as shown in Figure 2, unlike the AlexNet network, the output of MSFCN consists of three branches, as shown in Formula (1). The output dimension of $b^{\left(i\right)}(n=0,1,2)$ of each branch is set to 2048.

(1) $$F1=(b^{\left(0\right)}+b^{\left(1\right)}+b^{\left(2\right)})$$

After each pooling layer of the AlexNet network, a branch is added, and each branch is composed of a 1 × 1 convolution layer and a GAP layer. The 1 × 1 convolution layer is equivalent to a cross channel parameter pooling layer, which allows cross channel information interactive learning and helps to extract correlation features of different channels. GAP layer has the purpose of concatenating different feature mapping to reduce the parameters, and the output $b^{\left(i\right)}$ of each layer represents the feature mapping of each branch.

Suppose that the b−th(b=0,1,2) branch has $K^{\left(b\right)}$ channels $x_{k}^{\left(b\right)}|(k=1,2,\ldots ,K^{\left(b\right)})$, then the output of 1 × 1 convolution at the b−th branch is:

(2) $$y_{l}^{\left(b\right)}=\sum_{k=1}\omega_{kl}^{\left(b\right)}*x_{k}^{\left(b\right)},l=1,2,\cdots ,L$$

In Formula (2), l represents the number of output channels, which are set to 96, 256 and 256 among the three branches.

For the initialization of the MSFCN network, in order to maintain AlexNet’s good ability for emotion recognition, the main part of the MSFCN network is initialized by the trained AlexNet network parameters. For convolution of 1 × 1 of three branches, relevant parameters are generated randomly.

### 2.3. Bi-LSTM

In most cases, speech emotion signals have different durations, and LSTM model can deal with the features of variable length. As shown in Formulas (3)–(5), the LSTM network controls the network output through three “Gates”. Formulas (3)–(5) represent input Gate, forgetting Gate and output Gate respectively, in which the input gate determines the capacity of the input value, the forgetting gate determines the degree of forgetting the previous cell state, and the output gate determines the final output value. i, f, o, and *t* represent the input, forgetting, output, and time states respectively, while σ are sigmoid functions.

(3) $$f_{t}=\sigma (W_{f}[C_{t-1},h_{t-1},x_{t}]{+b}_{f})$$

(4) $$i_{t}=\sigma (W_{i}[C_{t-1},h_{t-1},x_{t}]{+b}_{i})$$

(5) $$o_{t}=\sigma (W_{o}[C_{t-1},h_{t-1},x_{t}]{+b}_{o})$$

Compared with LSTM, Bi-LSTM can learn speech features in both forward and backward directions, the deep features learned by Bi-LSTM are more robust than those learned by LSTM. Therefore, Bi-LSTM is used to extract frame emotional information in this paper.

### 2.4. Multi-Stream Sub-Branches Full Convolution Network

**Figure 3:**
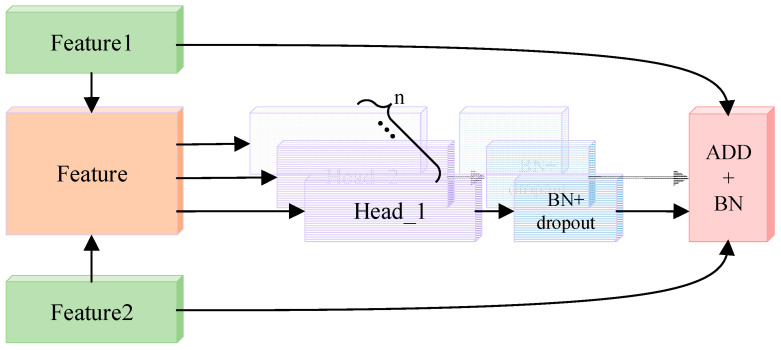
Multi-head attention mechanism.

In order to ensure the effect of feature fusion, this paper proposed a feature fusion method based on the multi-head attention mechanism [23]. As shown in Figure 3, considering the continuity of speech and the close connection between the upper and lower frames of speech, this paper uses all the feature frames in each attention mechanism header to avoid the loss of context sensitive information of speech features.

Taking i-head algorithm in the multi-head attention mechanism fusion algorithm as an example, we can use the attention weight of each dimension to express feature contribution. Specifically, the feature F obtained by splicing the deep features F1 and F2 output by different modules is recorded as [B, 2, N], 2 represents two different feature spaces, and N represents the size of feature dimension. In order to facilitate the calculation, same as ref. [19], this paper uses the self-attention mechanism algorithm to calculate the attention weight.

(6) $$\alpha_{i}^{F}=\mathrm{softmax}(\tanh(F\times W_{i}^{F})\times V_{i}^{F})$$

where $W_{i}^{F},V_{i}^{F}\in R^{N\times N}$ are the trainable attention mechanism parameters. The weighted output calculation method of feature $F^{\prime }\in \left[B,1,N\right]$ is shown in Formula (7).

(7) $$F^{\prime }={\mathrm{Attention}}_{i}(F1,F2)=\sum \alpha_{i}^{F}\times F$$

As shown in Formula (8), the input of each head in the multi-head weighted layer is calculated by the attention mechanism. Multi-header weighted processing increases the redundancy of information. As shown in Formula (9), dropout is added to the output layer of each header to eliminate redundant information. Then, the features of a different head are fused by summation.

(8) $$head_{i}={\mathrm{Attention}}_{i}(F1,F2)$$

(9) $$head_{i}{}^{\prime }=\mathrm{Dropout}(head_{i})$$

(10) $$Mulitihead(F1,F2)=\mathrm{Add}(head_{1}{}^{\prime },\ldots ,\;head_{n}{}^{\prime })$$

The original feature outputs of the two networks have their own feature details. However, the fusion features will lose these feature details after the dropout processing. At the same time, the multi-head operation presents the problem of gradient divergence. Therefore, as shown in Formula (11), in order to avoid the problems of slow model convergence or even non-convergence and over-fitting, the initial output of different networks is connected with the output of multi attention feature fusion layer through a shortcut connection.

(11) output=Add(F1,F2,Mulitihead(F1,F2))

Through the multi-head attention mechanism fusion, the feature output of the two networks belonging to different feature spaces will be concentrated in a new feature space, and the features that contribute a lot to emotion recognition will be given a greater weight factor, so the extracted fusion features are more representative.

## 3. Experiments

### 3.1. Speech Emotion Database

In order to show the performance of the proposed algorithm, this paper tests on the following three databases: CASIA database [24], EmoDB database [25], and SAVEE database [26]. The reasons for selecting these three databases are: Firstly, the languages of the three databases are Chinese, German, and English, which is helpful to exclude the influence of languages on the performance of the algorithm. Secondly, the three databases are public libraries, which is conducive to the algorithm reproduction.

CASIA database: The database is recorded by the Institute of automation, Chinese Academy of Sciences. It is a Chinese speech emotion database. The database contains 6 different emotions recorded by 4 actors (2 men and 2 women), including: anger, feel, happy, neutral, sad and surprise. The database used in this paper contains 1200 speeches, 300 speeches for each person and 50 speeches for each emotion.

EMODB is a German emotion database, by Berlin University. The library is composed of 10 actors (5 men and 5 women). The Emo-DB database used in this paper contains 535 sounds, including seven emotion types: anger (127), border (81), trouble (46), fear (69), happiness (71), neutral (79), and sadness (62). There are 49, 58, 43, 38, 55, 35, 61, 69, 56, 71 speeches for each person.

SAVEE is recorded by the University of Surrey. It is an English database. The database is recorded by four researchers. The SAVEE database used in this paper contains 480 speeches, including seven emotions, namely anger (60), trouble (60), fear (60), happiness (60), sadness (60), surprise (60), and neutral (120).

### 3.2. Evaluation Method

In this paper, we set up two different experiments to verify the effectiveness of the proposed algorithm: (1) In all databases, 80% of the data are randomly selected as the training set, and 20% of the data are selected as the test set. (2) This paper adopts the leave one speaker out (LOSO) strategy [24,27] to carry out the experiment. In this strategy, the data of one person are selected as the test set each time, the corpus of the remaining people is used as the training set, and the corpus of each person is used as the test set in turn to get the experimental results. Finally, the average value of all the experimental results is calculated as the final result.

Evaluation criteria: considering that the database data is unbalanced, weighted average recall (WA) and unweighted average recall (UA) [28] commonly used in the world are adopted as evaluation criteria. WA is the ratio of correctly identifying the number of test samples to the total number of all test samples. UA is the number of correctly identified test samples of each class divided by the number of test samples of each type. Finally, the average value of UA is obtained.

### 3.3. Experimental Parameters

Table 1 shows the specific parameters of MSCRNN-A. The MSCRNN-A used in this paper is built based on the tensorflow framework. The MSFCN network is initialized by AlexNet trained in Imagenet [29], and the output feature is 2048 dimension. Bi-LSTM consists of one hidden layer, and the output feature is 2048 dimension. In the multi-head fusion, the number of head of attention mechanism is set to 16, and dropout parameter is set to 0.5. The model parameters are optimized by minimizing cross entropy objective function. In order to prevent the model from failing to converge, we follow the settings of most literature [16,17,20], use Adam optimizer, and set the initial learning rate to 0.00001. In Table 1, B represents the batch size for each iteration, and L each dataset frame size.

### 3.4. Experimental between MSFCN and AlexNet

First, we use experimental strategy (2) to compare the traditional AlexNet with our MSFCN model. Through 200 epoch iterative training, the experimental results are shown in Table 2. From Table 2, we can see that the MSFCN model has made an obvious improvement. The experimental results in three databases show that the values of WA increased by 1.50%, 3.79%, and 1.25%, respectively, and the values of UA increased by 1.50%, 5.72%, and 2.03%, respectively. This shows that the ability of feature representation decreases with the deepening of network layers. MSFCN uses a branch structure to reduce the loss of emotional information, as well as more orderly feature representation to obtain higher performance.

### 3.5. Algorithm Comparison Experiment

This experiment mainly adopts an experimental strategy (1) to demonstrate the performance of the proposed MSCRNN model and fusion algorithm. The comparison algorithms include: (1) the PCRN algorithm used in reference [20]. (2) The MSCRNN model proposed in this paper uses splicing method to realize feature fusion. (3) MSCRNN-SA represents the MSCRNN model proposed in this paper and the single head attention mechanism feature fusion method used in reference [18]. (4) MSCRNN-A represents the model proposed in this paper.

**Figure 4:**
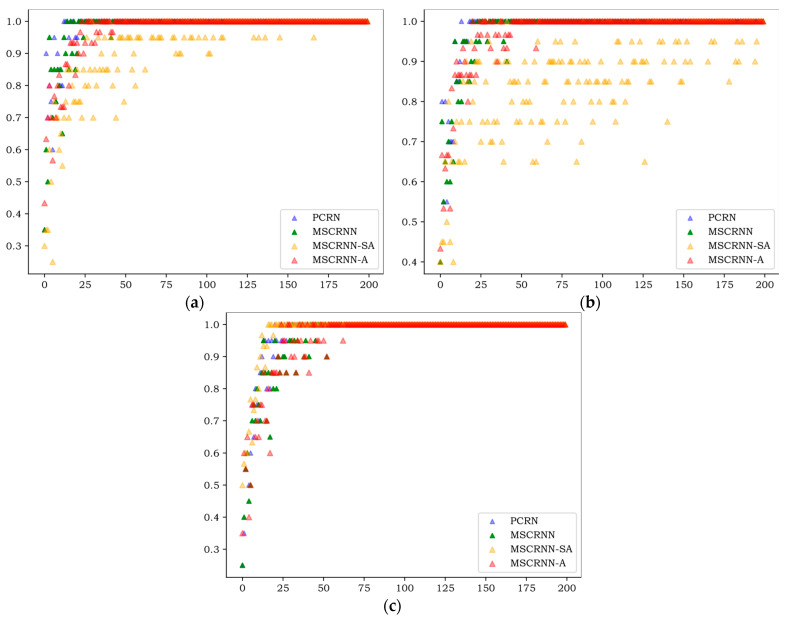
Convergence curves of training sets of different algorithms. (**a**) convergence curves of CASIA sets; (**b**) convergence curves of EMODB sets; (**c**) convergence curves of SAVEE sets.

**Figure 5:**
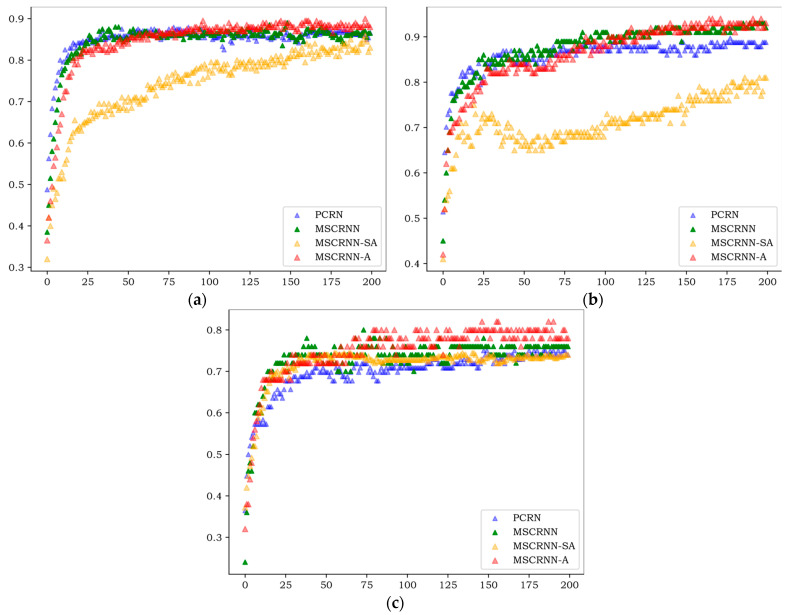
Recognition curves of test sets of different algorithms. (**a**) recognition curves of CASIA sets; (**b**) recognition curves of EMODB sets; (**c**) recognition curves of SAVEE sets.

**Figure 6:**
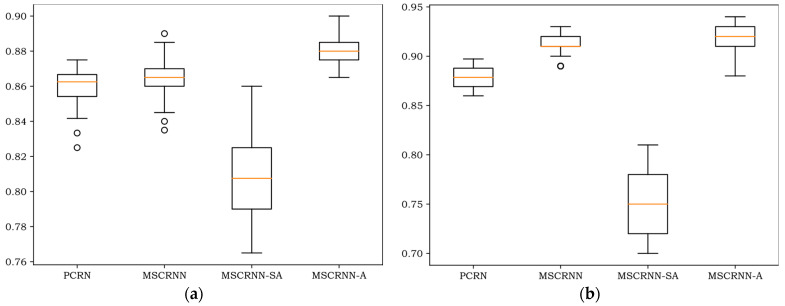

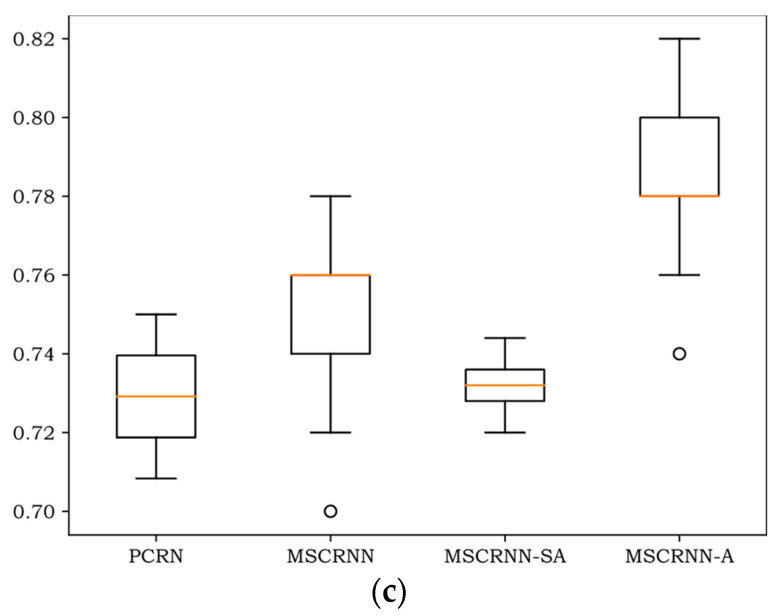
Box-plots of different databases. (**a**) Box-plot of CASIA database; (**b**) Box-plot of EMODB database; (**c**) Box-plot of SAVEE database.

Figure 4, Figure 5 and Figure 6 show the WA values of 200 rounds of four different algorithms on three databases. Figure 4 shows the convergence curves of training sets on three databases, and Figure 5 shows the recognition curves of test sets on three databases. Figure 6 shows the box diagram of WA values after 100 rounds in three libraries.

As can be seen from Figure 4, on the three databases, the curves of PCRN, MSCRNN, and MSCRNN-A begin to converge in 80 rounds, while MSCRNN-SA converges only after 170 rounds on CASIA, but not on EMODB. From the experimental results, we can see that MSCRNN-SA has the problem of gradient divergence. In MSCRNN-A, we connect the initial output of different modules with the output of the multi attention feature fusion layer through a shortcut connection to avoid this problem.

As can be seen in Figure 5, the results of the test set are similar to those of the training set in Figure 4, and the algorithm begins to converge after 80 rounds. On the three databases, the recognition rate of PCRN and MSCRNN-SA is significantly lower than that of MSCRNN and MSCRNN-A. Compared with PCRN, MSCRNN uses MSFCN and Bi-LSTM to extract emotional features. MSFCN reduces the loss of emotional information in deep convolution network. Bi-LSTM is composed of bidirectional LSTM, which can obtain more emotional information. Therefore, MSCRNN has better recognition performance than PCRN. In the MSCRNN-SA algorithm, although reference [12] points out that it can achieve good performance in classification, it can be seen from Figure 4 that in CASIA and EMODB databases, the model suffers from the problem of gradient divergence, which makes the recognition performance poor. Compared with MSCRNN and MSCRNN-A algorithm, it can be seen from Figure 5 that, on the three databases, the red curve representing MSCRNN-A is mostly at the top, and the best recognition results are obtained. Especially on the SAVEE and CASIA databases, the recognition performance of MSCRNN-A algorithm is better than the other three algorithms after 100 rounds.

As can be seen in Figure 6, notwithstanding that the minimum of MSCRNN-A is less than MSCRNN on EMODB, all the maximum, minimum, 75th-percentile, 25th-percentile, and median values of MSCRNN-A are better than other algorithms on the three databases. Compared with CRNN algorithm, the median value of MSCRNN-A is improved by 1.5%, 1%, and 2% respectively on CASIA, EMODB, and SAVEE. The recognition performance is significantly better than the other three algorithms.

### 3.6. Compare to State-of-the-Art

Firstly, this paper uses the ComParE feature set and SVM algorithm to construct the emotion recognition system as the baseline of the comparison algorithm. The ComParE feature set is widely used around the world [13,30], containing some 6373 dimensional features.

Secondly, we compare the proposed algorithm with some of the most advanced algorithms. The comparison algorithm includes: HuWSF features used in reference [7]; RDBN network used in reference [31], PCRN network used in reference [20], DCNN-DTPM feature used in reference [9], and 3D ACRNN used in reference [18].

Table 3 shows the comparison results obtained adopting experimental strategy (2) between MSCRNN-A and other algorithms. Firstly, compared with baseline, the WA/UA of MSCRNN-A is 14.67% higher than baseline on CASIA. On EMODB database, the WA of MSCRNN-A is 5.3% higher than baseline, and the UA is 7.78% higher than baseline. On the SAVEE, the WA of MSCRNN-A is 6.25% higher than baseline, and the UA is 7.17% higher than baseline. On three databases, the performance of MSCRNN-A is significantly better than that of baseline algorithm.

Compared with HuWSF, RDBN, and PCRN, the WA/UA of MSCRNN-A is in-creased by 17.25%, 12.25%, and 2.5% in CASIA. On EMODB, compared with DCNN-DTPM, PCRN, and 3D ACRNN, the UA values of the proposed algorithm are improved by 5.13%, 3.42%, and 1.65% respectively, while compared with DCNN-DTPM and PCRN algorithm, the WA value increased by 1.1% and 1.97% respectively. On the SAVEE database, compared with PCRN, the UA value of MSCRNN-A is increased by 6.22%. Compared with HuWSF, RDBN, and PCRN, the UA of MSCRNN-A is increased by 20.83%, 12.65%, and 3.76% respectively. On different databases, the proposed algorithm performs better than the state-of-the-art algorithm.

**Figure 7:**
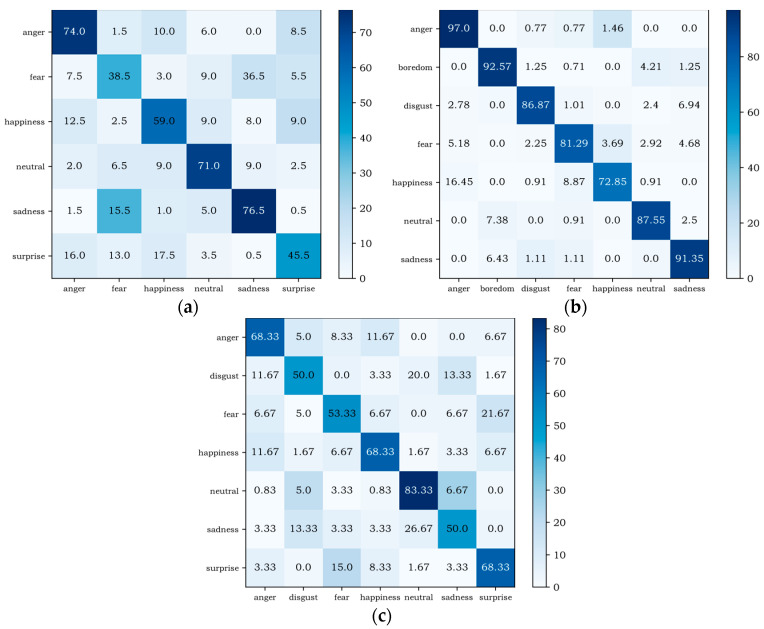
Confusion matrixs of different databases. (**a**) CASIA database confusion matrix; (**b**) EMODB database confusion matrix; (**c**) SAVEE database confusion matrix.

Figure 7 shows the confusion matrix of MSCRNN-A on different databases. From the experimental results of the three databases, it can be seen that the recognition performance of some emotions is similar on the three databases. For example, the recognition rates of anger and neutral are relatively high, and anger emotions are easily misrecognized as happy. However, there are also many differences. For example, on EMODB and CASIA, the recognition performance of sadness is higher, but on SAVEE, the recognition performance of sadness is relatively lower. The reasons for these differences are as follows: Firstly, the emotion types of the three databases are different, and the feature space of some emotion types on some databases is close. Secondly, different languages and different cultural backgrounds make the pronunciation different. Finally, the sample size is different, because the various emotion types of CASIA database are consistent, while the sample numbers of EMODB and SAVEE are not consistent, so the recognition of different emotions in network training models presents certain differences, eventually leading to certain differences in the recognition performance of related emotions in different databases.

## 4. Summary

This paper presents a multi-stream convolution-recurrent neural networks based on attention mechanism (MSCRNN-A) for speech emotion recognition. Firstly, the algorithm prevents the increase of emotional information entropy and the loss of emotional information by adding branches, and then proposes a feature fusion model based on the multi attention mechanism. Thirdly, the initial output of different modules is connected with the output of a multi attention feature fusion layer obtained using a shortcut connection to suppress the degradation of network capability caused by gradient divergence. The effectiveness of the proposed algorithm is verified in the simulation phase.

## Figures and Tables

**Table 1:** MSCRNN parameters.

Module	Layer	Shape
MSFCN	Input1	B × 227 × 227 × 3
	Conv1	11 × 11 × 96
	1 × 1 Conv_1	1 × 1 × 2048
	Conv2	5 × 5 × 256
	1 × 1 Conv_2	1 × 1 × 2048
	Conv3	3 × 3 × 384
	Conv4	3 × 3 × 384
	Conv5	3 × 3 × 256
	1 × 1 Conv_3	1 × 1 × 2048
	Output1	B × 2048
Bi-LSTM	Input2	B × 64 × L
	Hidden units (FW)	FW:2048
	Hidden units (BW)	BW:2048
	Output1	B × 2048
Output		B × 4096

**Table 2:** Performance comparison between MSFCN and AlexNet.

DATABASE	Algorithm	WA	UA
CASIA	AlexNet	48.66%	48.66%
	MSFCN	**50.16%**	**50.16%**
EMODB	AlexNet	76.50%	71.86%
	MSFCN	**80.29%**	**77.58%**
SAVEE	AlexNet	56.87%	52.85%
	MSFCN	**48.12%**	**54.88%**

**Table 3:** Performance comparison with state-of-the-art.

DATABASE	Algorithm	WA	UA
CASIA	Baseline	46.08%	46.08%
	HuWSF	41.92%	41.92%
	RDBN	48.5%	48.50%
	PCRN	58.25%	58.25%
	Proposed Algorithm	**60.75%**	**60.75%**
EMODB	Baseline	83.11%	80.17%
	DCNN-DTPM	87.31%	86.30%
	RCRN	86.44%	84.53%
	3D ACRNN	-	82.82%
	Proposed Algorithm	**88.41%**	**87.95%**
SAVEE	Baseline	60.00%	58.45%
	HuWSF	45.42%	-
	RDBN	53.60%	-
	PCRN	62.49%	59.40%
	Proposed Algorithm	**66.25%**	**65.62%**
